# [Retracted] Circular RNA NOX4 promotes the development of colorectal cancer via the microRNA‑485‑5p/CKS1B axis

**DOI:** 10.3892/or.2024.8780

**Published:** 2024-07-22

**Authors:** Ximin Wang, Geng Tao, Donghong Huang, Shuangyin Liang, Dongxu Zheng

Oncol Rep 44: 2009–2020, 2020; DOI: 10.3892/or.2020.7758

Following the publication of the above paper, it was drawn to the Editor's attention by a concerned reader that there appeared to be a matching data panel comparing between one of the Transwell invasion assay experiments (the ‘SW620/si-NC’ data panel) shown in Fig. 2F and Fig 6D in the following paper, written by different authors at different research institutes, that had already been published at the time of this paper's submission: Wang D, Yang T, Liu J, Liu Y, Xing N, He J, Yang J and Ai Y: Propofol inhibits the migration and invasion of glioma cells by blocking the PI3K/AKT pathway through miR-206/ROCK1 axis. Onco Targets Ther 13: 361–370, 2020. In addition, a potential problem regarding the design of the experiment was noted with the selection of the primers for the amplification of the miRNA miR-485-5p.

Owing to the fact that the contentious data in the above article had already been published prior to its submission to *Oncology Reports*, the Editor has decided that this paper should be retracted from the Journal. The authors were asked for an explanation to account for these concerns, but the Editorial Office did not receive a reply. The Editor apologizes to the readership for any inconvenience caused.

